# Gout and Hay-Fever

**Published:** 1900-07-14

**Authors:** 


### GOUT AND HAY-FEVER.

Writing on the subject of hay-fever, Dr. Karl Grube,[1] of Neuenahr, insists on its connection with the gouty state. He says that in most of his cases, and in all the cases which he treated successfully, a gouty tendency could be traced underlying the local affection, the patients either coming of a gouty stock, without themselves having any other symptoms of gout, or suffering from gout in some form or other. Strictly speaking, he says, the universally-used term "hay-fever" is misleading, for the catarrh is not produced only by grass or hay, nor is it entirely limited to the summer months. It is also to be noted that persons who suffer from hay-fever will often be found to suffer more or less freely from catarrhs at other times of the year, only the attacks are more frequent and more severe during the summer, such cases being examples of that general tendency to catarrh of the mucous membranes so frequently to be observed in gouty subjects. The treatment which he recommends is a careful and strict regulation of the diet on lines directed against the gouty tendencies, and locally the application of the Neuenahr waters in the form of inhalations, gargles, and nose-douches. We cannot send all our patients to Neuenahr, but we may take the hint. Certainly in the treatment of hay-fever considerable advantage is gained by the use of weak alkaline and saline nasal douching; and, although at Neuenahr they use the Neuenahr water (which is a simple saline, at a temperature of 96 deg. Fahr., containing 0·6 per mille of carbonate of sodium, 0·2 per mille of carbonate of calcium, and 0·3 per mille of carbonate of magnesium), it is not difficult to produce a very useful substitute, while as to the gouty regimen it ought to be as easy to give it in one place as another, if only patients would follow the directions given.

[1] Lancet, July 7.

